# Blood Donation in Lebanon: A Six-Year Retrospective Study of a Decentralized Fragmented Blood Management System

**DOI:** 10.7759/cureus.21858

**Published:** 2022-02-03

**Authors:** Jules-Joel Bakhos, Myra Khalife, Yorgui Teyrouz, Youakim Saliba

**Affiliations:** 1 Department of Medical Research, Donner Sang Compter (DSC), Beirut, LBN; 2 Faculty of Medicine, Saint Joseph University, Beirut, LBN

**Keywords:** lebanon, developing world, fragmented blood system, blood type prevalence, blood donation

## Abstract

Introduction

In developing countries, the lack of a sufficient and safe blood supply is a significant impediment to providing health care. Lebanon is notable for its absence of a Donor Management System to ensure continuous donor recruitment and scheduling. Herein, we report the findings of Lebanon's first large retrospective population-based study to investigate blood types and donation that is critical for managing community blood supply.

Methods

The non-remunerated voluntary blood donors were recruited by the non-profit organization “Donner Sang Compter”. The study spanned six years, from August 2015 to May 2021, and included 36,002 people from 18 districts throughout Lebanon's nine governorates.

Results

The most prevalent blood type was A (42%), followed by O (37.48%), B (13.86%), and the AB group (6.84%). RhD+ groups were predominant (88.45%), with A+ being the most (37.84%) and AB- being the least prevalent (1.05%). Furthermore, blood type and donation profiling revealed a substantial geographical variation in the frequency of blood groups, despite the relatively small country’s area. As for blood donation, when gender and age were considered, young male donors dominated the pool across the country.

Conclusion

This study on blood type prevalence and blood donor demographics may pave the way for the development of a more coherent and integrated blood management system in Lebanon, as opposed to the fragmented and decentralized system now in existence. These findings also provide crucial clinical information for the country's future transfusion medicine policies and practices, which is vital in such a precarious part of the world.

## Introduction

A sufficient supply of blood transfusions is required for a well-functioning healthcare system. Even though blood and its components were recently included in the World Health Organization's (WHO) Model List of Essential Medicines, inadequate blood inventories continue to contribute to preventable death and morbidity in many countries [1, 2]. In 2017, a modeling study estimated that the world's blood demand exceeded 300 million units, while the world's blood supply fell short by 30 million blood product units. The result is also a scarcity of safe and adequate blood supplies in poor and middle-income nations, with the causes of these shortages being multifactorial [3-5]. Consequently, the lack of an adequate and safe blood supply is a significant impediment to health care in developing countries [1, 5].

Blood group prevalence studies are crucial to managing blood product supplies in the community [6]. The distribution of ABO and RhD blood groups varies throughout the world [7, 8]. Although the ABO blood type antigens remain stable throughout life, the distribution of blood groups and, more crucially, blood donors across various communities, ethnic groups, and geographical borders do change over time, even within the same geographic area [9]. Therefore, it is crucial to keep track of the continuous changes in the predominance of different blood types in the general population.

According to the WHO, all operations connected to blood collection, testing, processing, storage, and distribution should be coordinated at the national level via efficient organization and integrated blood supply networks [4]. Nonetheless, Lebanon features a decentralized/fragmented blood transfusion system, with the majority of blood donations taking place in hospitals, and a minor proportion taking place at nongovernmental organizations [2, 10-13]. Likewise, in the Middle East, each country has its own blood transfusion system and blood donation practices differ from one country to another [14].

In this paper, we conducted the first large retrospective population-based study over six years to investigate the distribution of ABO and RhD blood groups and characterize blood donors in the general population in Lebanon to provide reliable data for better development of rational strategies for blood collection and management in a fragmented decentralized blood system.

## Materials and methods

Donor recruitment

The blood donors were recruited by the non-governmental non-profit association “Donner Sang Compter” (DSC) that was founded in 2010 with the sole goal of establishing a network of willing volunteers who would freely donate blood at any time of year. To maintain privacy and anonymity and to prevent social or financial pressure, donor names were kept utterly confidential via the contact center. Social media was used to recruit young prospective donors. In addition, during the year, many blood drives were held in collaboration with hospital blood banks in various public locations, including colleges, shopping malls, and corporate locations, to boost inventory. In order to debunk some of the numerous misunderstandings regarding blood donation in Lebanon, awareness campaigns and events were held in various public spaces. In contrast to the replacement donors, DSC donors were genuine non-remunerated volunteers who gave anonymously and were solely driven by the donation experience.

Study population characteristics and blood collection

The research was conducted in a retrospective manner over six years, from August 2015 to May 2021. The DSC database was used to compile the yearly statistics on nationwide population-based blood donors, which were then collected into this report. The research comprised 36,002 participants from 18 districts throughout Lebanon’s nine governorates; 23,493 (65.2%) were male, and 12,509 (34.8%) were female. Participants were divided into two sections, those willing to donate but had never done this before and those who are proven donors. To determine the prevalence of different blood types throughout Lebanon’s districts and governorates, the total number of participants was considered. Proven donors or “donors” were defined as individuals who donated at least once during the study’s six-year period. In total, 19,048 participants donated at least once, with 10,494 (55.09%) donating just once and 17,485 (91.79%) donating no more than five times. Male donors represented the vast majority of donors and were 14,530 individuals accounting for 76.28%, whereas female donors were 4,518 individuals accounting for 23.72% of all donors (Table 1).

**Table 1:** Demographic characteristics of the study population.

	Total (%)
Male participants	23,493 (65.2%)
Female participants	12,509 (34.8%)
Male donors	14,530 (76.28%)
Female donors	4,518 (23.72%)
Age 18-25	6,892 (36.18%)
Age 26-45	10,306 (54.11%)
Age 46-65	1,806 (9.48%)

The Test Tube method was used to identify blood samples after collection at the regional blood banks throughout Lebanon, whereby one drop of 5% isotonic saline suspending red blood cells was added to Anti-A and Anti-B antigens that were present in a clean, labeled test tube. ABO blood type system screening comprised testing red cells with Anti-A and Anti-B antibodies and testing the serum with a mixture of A and B red blood cells. In the case of Rhesus D antigen (RhD), the Anti-D reagent was employed to detect whether the blood sample was positive or negative for Rhesus.

Statistical analysis

Statistical analysis was conducted using GraphPad Prism 9 (GraphPad Software, San Diego, CA, USA). D’Agostino and Pearson’s normality test was used to check whether populations followed a Gaussian distribution. Then, to test whether populations had equal variances, Bartlett’s test was used when Gaussian distribution was present, whereas Brown-Forsythe test was used when the data were skewed. For comparisons of greater than two groups, and when Gaussian distribution with unequal variances was met, Brown-Forsythe and Welch ANOVA tests were applied. When data were skewed, Kruskal-Wallis One-Way ANOVA on Ranks was used. When comparing two-sample groups, an unpaired two-tailed T-test was used when Gaussian distribution with equal variances was met. When data were skewed, a non-parametric two-tailed Mann-Whitney test was performed. The Chi-Square Test of Independence was used to determine whether there is an association between blood groups and donors, age, sex, and the districts. *P* values less than 0.05 were considered significant.

## Results

Demographic characteristics of the study population

**Figure 1:**
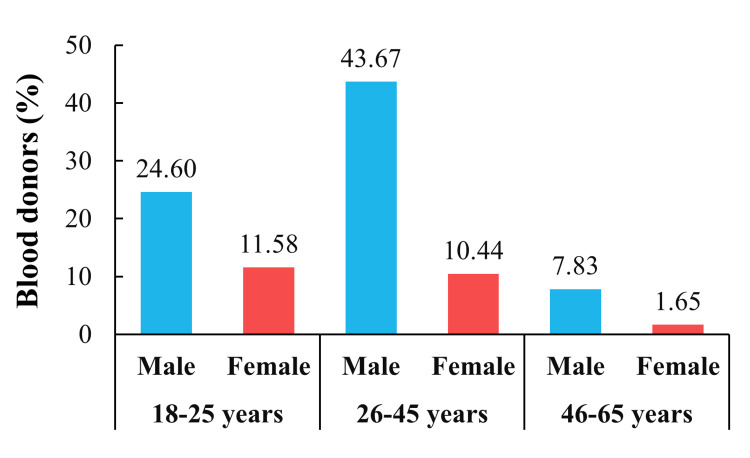
Blood donor distribution across gender and age in Lebanon. The proportions of male and female donors in each age interval are reported. Interaction of gender and age was tested using Chi-square test (X^2^ = 3.189; *p* = 0.203).

Donors between the ages of 26 and 45 tended to be the most common (54.11%), followed by donors between the ages of 18 and 25 (36.18%), and then donors between the ages of 46 and 65 (9.48%). In addition, male donors between the ages of 26 and 45 tended to be the most prevalent, followed by those aged between 18 and 25, then female donors between 18 and 25, those between 26 and 45, and lastly male and female donors between 46 and 65. Thus, young male donors were the most prevalent (68.27%). However, the Chi-square test did not reveal any statistical significance in the gender and age interaction (X^2^ = 3.189; *p *= 0.203) (Figure 1).

General prevalence of ABO and RhD blood groups in Lebanon

Upon considering the ABO without the RhD system, blood group A appeared to be the most frequent, followed by the O group, then B group, and finally the AB group being the least common (Table 2). Upon considering both ABO and RhD systems, RhD+ groups were more prevalent, with A+ being the most frequent, followed by O+, B+, and AB+. RhD- groups were less prevalent in the following order, A-, O-, B-, and AB-. In total, RhD+ groups were predominant over RhD- groups, accounting for 88.45% against 11.55% (Table 2).

**Table 2:** General distribution of ABO and RhD groups in Lebanon.

ABO and RhD phenotypes		Total (%)	Total (%)
A	A+	13,623 (37.84)	15,122 (42)
	A-	1,499 (4.16)	
B	B+	4,304 (11.95)	4,919 (13.86)
	B-	615 (1.71)	
AB	AB+	2,086 (5.79)	2,466 (6.84)
	AB-	380 (1.05)	
O	O+	11,831 (32.86)	13,495 (37.48)
	O-	1,664 (4.62)	
RhD+			31,844 (88.45)
RhD-			4,158 (11.55)

Blood donor distribution across ABO groups and gender

**Figure 2:**
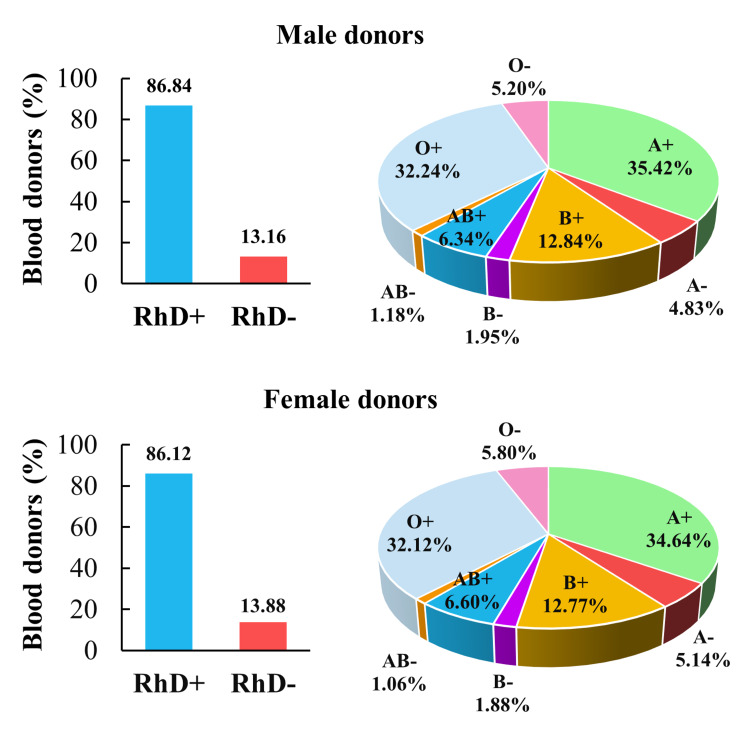
Distribution of blood donors among ABO blood types and gender in Lebanon. The proportions of male and female donors, according to their respective blood groups, are reported. The statistical difference between female and male donors’ blood types distribution was evaluated using the Chi-square test (X^2^ = 0.1256; *p* = 0.999).

The act of donating blood at least once throughout the research period was the only criteria for blood donors to be considered as such. In the ABO and RhD systems, upon considering the male gender, the A+ group was the most prevalent (5,147 donors or 35.42%), followed by O+ (4,684 donors or 32.24%), B+ (1,866 donors or 12.84%), AB+ (921 donors or 6.34%), O- (755 donors or 5.2%), A- (702 donors or 4.83%), B- (284 donors or 1.95%), and lastly AB- (171 donors or 1.18%). In total, male donors of the RhD+ phenotype were 12,618 donors or 86.84%, and those of the RhD- were 1,912 donors or 13.16% (Figure 2).

In female donors, comparable percentages were observed: A+ (1,565 donors or 34.64%), O+ (1,451 donors or 32.12%), B+ (577 donors or 12.77%), AB+ (298 donors or 6.6%), O- (262 donors or 5.8%), A- (232 donors or 5.14%), B- (85 donors or 1.88%), and AB- (48 donors or 1.06%) (Figure 2). In total, female donors of the RhD+ phenotype were 3,891 donors or 86.12%, and those of the RhD- were 627 donors or 13.88%.

Governorate distribution of blood groups and donors according to the ABO blood group system in Lebanon

**Figure 3:**
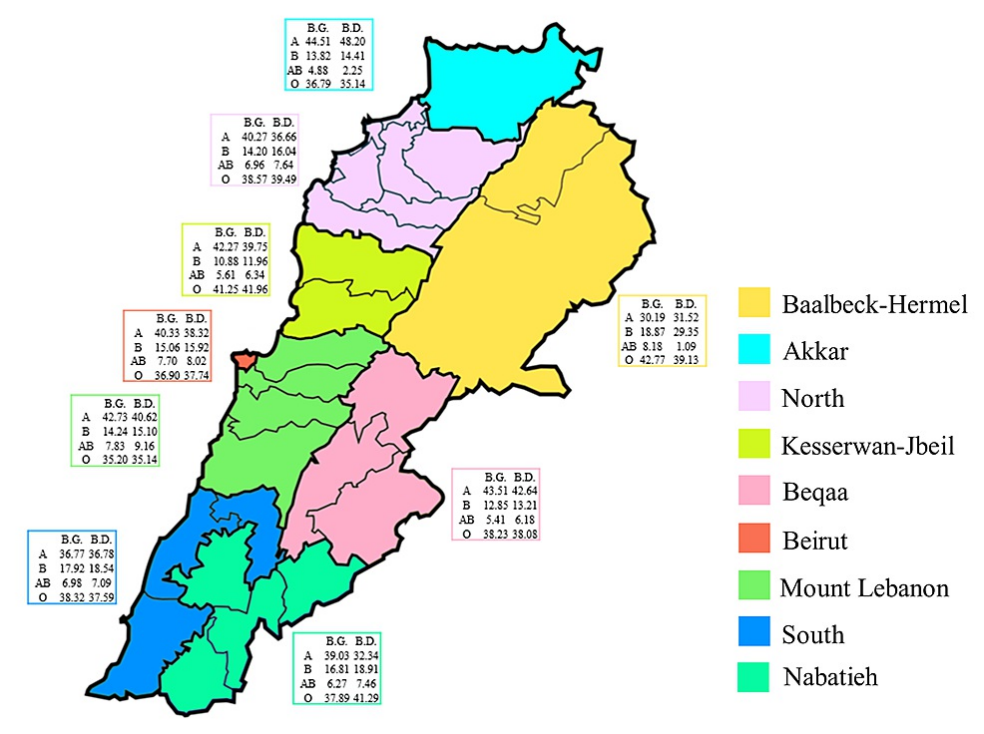
Distribution of blood groups (BG) and blood donors (BD) according to the ABO system and the different governorates in Lebanon. The proportions of BG and BD, based on the ABO system, are reported. The statistical difference in B.D. distribution between the various governorates was evaluated using the Chi-square test, X^2^ = 83.71, *p* = 0.0026, whereas the difference between the different B.D. ABO groups across the governorates was evaluated using Brown-Forsythe ANOVA, *p* < 0.0001. As for B.G. distribution, X^2^ = 24.47, *p* = 0.9994, and Brown-Forsythe ANOVA, *p* < 0.0001.

Blood donors’ distribution according to the ABO groups displayed a significant difference between the governorates (X^2^ = 83.71, *p* = 0.0026). The highest differences were noted in the A and B donor groups; the governorates of Nabatieh and Baalbeck-Hermel had the lowest proportions of group A donors, whereby the South, Nabatieh, and Baalbeck-Hermel had the highest proportions of group B donors (Figure 3). Prevalence of blood groups showed a similar pattern for group B, with the South, Nabatieh, and Baalbeck-Hermel having the highest proportions (Figure 3). However, no overall significance in blood groups’ prevalence according to the ABO groups was found between the governorates (X^2^ = 24.47, *p* = 0.9994). Among blood donors in all governorates, proportions (95% CI) were as follows: A (36.5-41.04), B (14.32-18), AB (5.241-7.857), and O (32.37-40.58). Among blood groups in all governorates, proportions (95% CI) were as follows: A (38.21-42.24), B (12.74-15.26), AB (5.487-6.957), and O (36.07-38.93). Finally, a significant difference was noted between the various blood donors' ABO groups across the governorates (*p* < 0.0001), with a similar pattern for blood groups (*p* < 0.0001); group A was the most prevalent amongst blood groups, whereas group O was predominant amongst donors (Figure 3).

Governorate distribution of blood groups and donors according to the RhD blood group system in Lebanon

**Figure 4:**
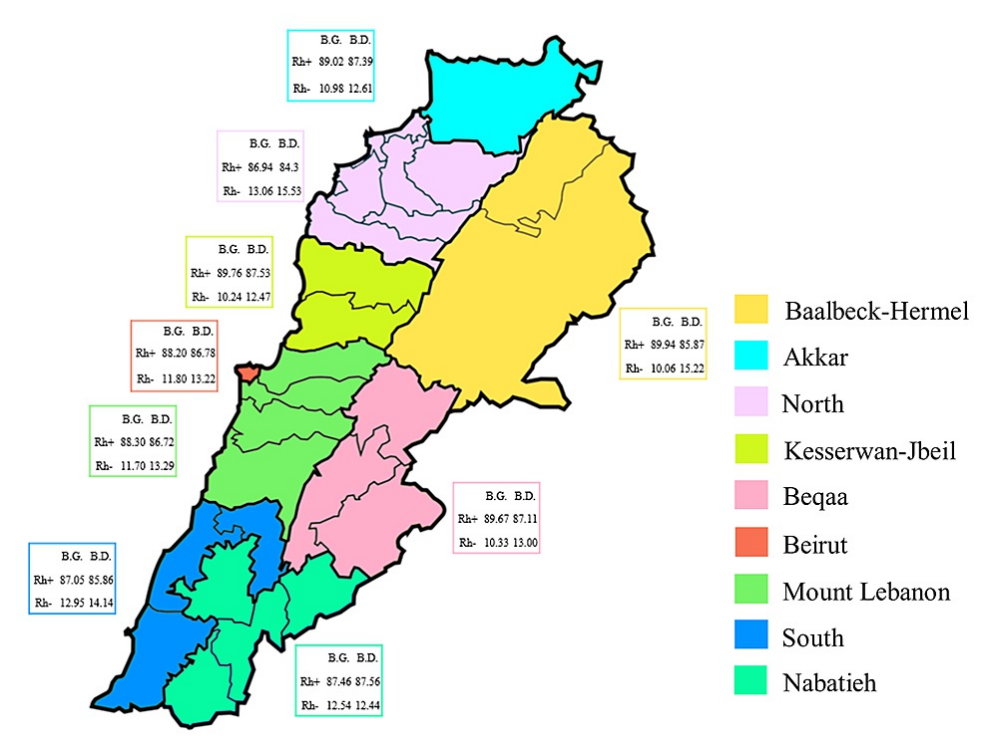
Distribution of blood groups (B.G.) and blood donors (B.D.) according to the RhD system and the different governorates in Lebanon. The proportions of B.G. and B.D., based on the RhD system, are reported. The statistical difference in B.D. distribution between the various governorates was evaluated using the Chi-square test, X^2^ = 15.52, *p* = 0.5581, whereas the difference between the B.D. ABO groups across the governorates was evaluated using the Mann-Whitney U test, *p* < 0.0001. As for B.G. distribution, X^2^ = 2.675, *p* > 0.9999, and unpaired two-tailed T-test, *p* < 0.0001.

Blood donors’ distribution according to the RhD phenotypes was comparable across the governorates (X^2^ = 15.52, *p* = 0.5581), and the same was noted for blood groups prevalence (X^2^ = 2.675, *p* > 0.9999) (Figure 4). Among blood donors in all governorates, proportions (95% CI) were as follows: RhD+ (79.38-88.18) and RhD- (12.59-14.75). Among blood groups in all governorates, proportions (95% CI) were as follows: RhD+ (87.21-88.45) and RhD- (10.55-11.79). Finally, a significant difference was noted between the various blood donors' RhD groups across the governorates (*p* < 0.0001), with a similar pattern for blood groups (*p* < 0.0001); RhD+ was predominant in all regions (Figure 4).

Governorate distribution of blood groups and donors according to the ABO and RhD blood group systems in Lebanon

**Figure 5:**
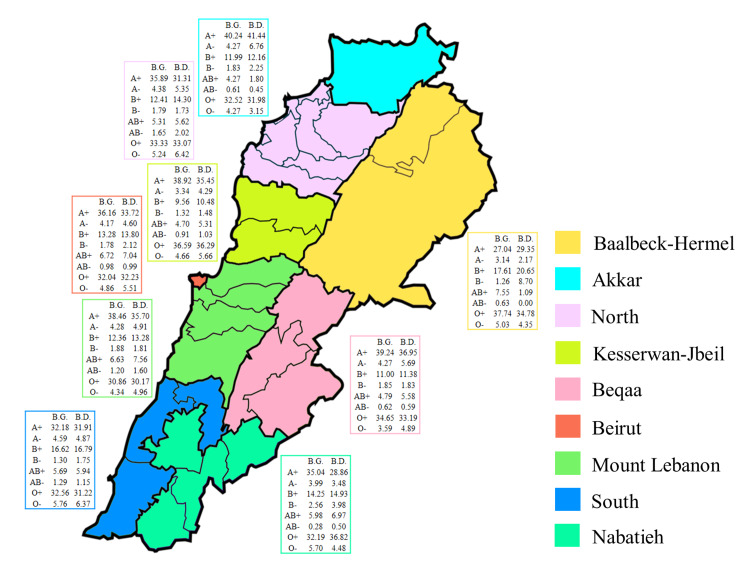
Distribution of blood groups (B.G.) and blood donors (B.D.) according to the ABO and RhD systems and the different governorates in Lebanon. The proportions of B.G. and B.D., based on the ABO and RhD systems, are reported. The statistical differences in B.D. and B.G. distribution across the governorates were evaluated using Kruskal-Wallis ANOVA on Ranks, and Brown-Forsythe and Welch ANOVA (*p* < 0.0001 for both tests).

Donor behavior and blood group distribution were not affected by geographical differences (Table 3, Figure 5). Noteworthy, higher proportions of B+ groups were present in the South, Nabatieh, and Baalbeck-Hermel (+37.33% in comparison to other regions), associated with lower proportions of A+ groups (-21.41% in comparison to other regions) (Table 3, Figure 5). In most governorates, the A+ group was predominant, followed by the O+ group, except for the South and Baalbeck-Hermel, where the O+ group was the most prevalent (Figure 5). The least prevalent blood group in all governorates was the AB- which was also reflected by the scarcity of its donors.

**Table 3:** Distribution of ABO RhD blood donors and groups in the study population according to Lebanon governorates.

B.G.	95% CI	Brown-Forsythe and Welch ANOVA
A+	34.12-37.88	P<0.0001
A-	3.31-4.13	
B+	10.89-13.66	
B-	1.04-1.50	
AB+	4.64-5.8	
AB-	0.19-0.8	
O+	31.61-34.16	
O-	3.851-4.816	
B.D.	95% CI	Kruskal-Wallis ANOVA on Ranks
A+	31.47-35.3	P<0.0001
A-	3.67-4.99	
B+	11.89-14.78	
B-	0.85-2.58	
AB+	4.15-6.17	
AB-	0.21-1.11	
O+	30.83-33.84	
O-	4.35-5.42	

Governorate distribution of blood donors according to gender in Lebanon

**Figure 6:**
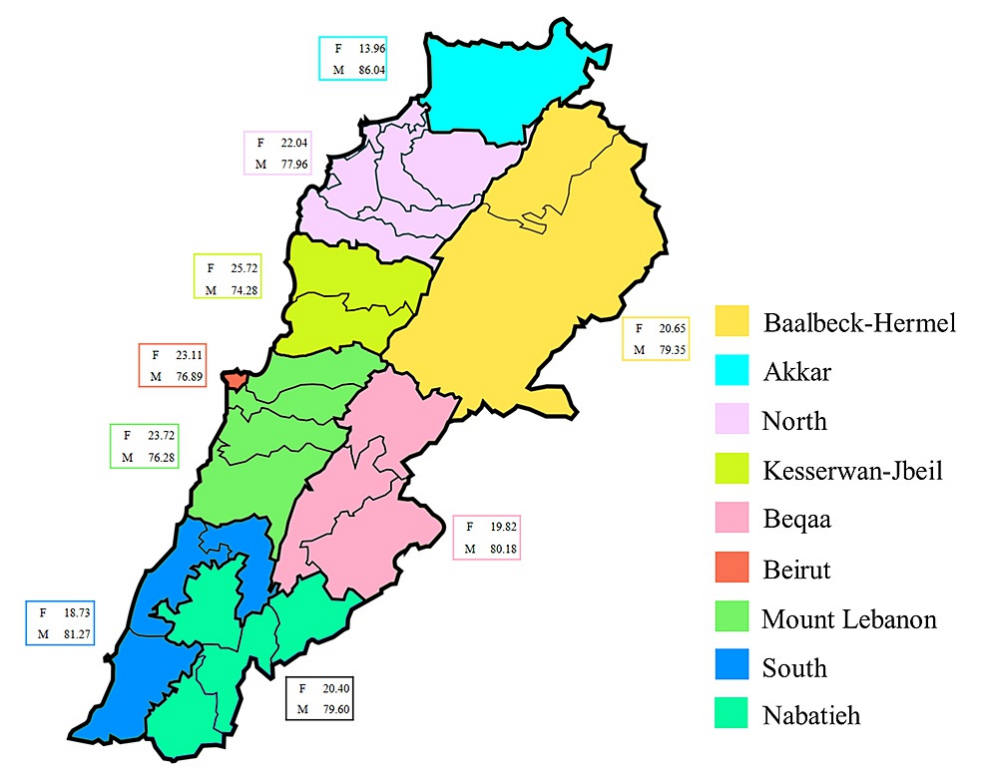
Lebanon's governorate distribution of blood donors by gender. The statistical differences in donor’s distribution were evaluated using the Chi-square test, X^2^ = 6.423, *p* = 0.6, whereas the difference in donor gender across all governorates was evaluated using an unpaired two-tailed T-test, *p* < 0.0001.

In all governorates, blood donors were divided into groups based on their gender. However, there was no statistically significant variation in the distribution of genders across the areas (X^2^ = 6.423, *p* = 0.6). The most empirical finding was that men constituted a much larger proportion of the donor population than women. The corresponding 95% CI were: Female (19.05-23.17) and Male (75.83-79.95) (*p* < 0.0001) (Figure 6).

District distribution of blood groups and donors in Lebanon

The frequency of blood types and the behavior of donors were observed in 18 districts across Lebanon. Certain districts emphasized the differences between the A and B groups; Tyre and Baalbeck, for example, had large percentages of B+, while the proportion of A+ was low in both cases. The blood type AB-, which is the least common, was most prevalent in Batroun and Koura (Table 4).

**Table 4:** District distribution of blood groups (B.G.) and donors (B.D.) according to the ABO and RhD blood group systems in Lebanon.

	A+		A-		B+		B-		AB+		AB-		O+		O-	
	B.G.	B.D.	B.G.	B.D.	B.G.	B.D.	B.G.	B.D.	B.G.	B.D.	B.G.	B.D.	B.G.	B.D.	B.G.	B.D.
Akkar	40.24	41.44	4.27	6.76	11.99	12.16	1.83	2.25	4.27	1.80	0.61	0.45	32.52	31.98	4.27	3.15
Aley	39.58	36.50	3.81	4.01	12.73	13.02	2.00	1.95	7.49	7.66	1.69	2.68	28.40	28.83	4.31	5.35
Baabda	38.04	36.97	4.11	4.67	12.74	13.61	1.73	1.81	6.04	6.89	1.16	1.34	31.49	29.79	4.69	4.91
Baalbeck	27.04	29.35	3.14	2.17	17.61	20.65	1.26	8.70	7.55	1.09	0.63	0.00	37.74	34.78	5.03	4.35
Batroun	33.33	28.57	2.10	2.65	10.96	13.23	1.86	2.65	4.90	4.76	2.80	2.65	37.30	39.15	6.76	6.88
Beirut	36.16	33.72	4.17	4.60	13.28	13.80	1.78	2.12	6.72	7.04	0.98	0.99	32.04	32.23	4.86	5.51
Beqaa	36.53	34.55	5.18	6.87	12.44	12.45	1.68	1.72	5.05	6.01	0.91	0.86	35.23	34.12	2.98	3.65
Chouf	36.39	32.00	4.76	5.60	13.72	15.60	2.04	1.60	7.71	9.60	0.91	1.40	30.50	29.80	3.97	4.40
Jbeil	38.10	34.66	3.10	3.84	9.43	10.09	1.18	0.99	5.38	5.68	0.96	0.99	36.77	38.07	5.08	5.68
Kesserwan	39.75	36.24	3.59	4.75	9.68	10.87	1.46	1.96	4.01	4.93	0.87	1.07	36.40	34.52	4.23	5.64
Koura	38.90	31.55	4.40	7.49	10.77	13.90	1.32	2.14	4.40	3.74	1.76	3.21	34.07	31.02	4.40	6.95
Metn	39.81	37.34	4.43	5.37	10.24	10.91	1.76	1.89	5.29	6.07	1.03	0.98	33.04	32.27	4.40	5.18
Nabatieh	35.04	28.86	3.99	3.48	14.25	14.93	2.56	3.98	5.98	6.97	0.28	0.50	32.19	36.82	5.70	4.48
Sidon	34.94	35.18	4.70	5.65	14.03	14.50	1.60	2.13	5.90	5.97	1.09	1.39	32.70	30.17	5.04	5.01
Tripoli	32.93	30.63	5.45	5.67	15.64	17.15	1.30	0.00	7.26	7.50	1.38	1.38	30.94	30.63	5.10	5.82
Tyre	29.43	28.64	4.49	4.09	19.20	19.09	1.00	1.36	5.49	5.91	1.50	0.91	32.42	32.27	6.48	7.73
Zahle	41.95	39.35	3.36	4.52	9.56	10.32	2.01	1.94	4.53	5.16	0.34	0.32	34.06	32.26	4.19	6.13
Zgharta	38.39	34.48	5.58	5.60	12.28	12.93	2.68	2.16	4.69	6.47	0.67	0.86	31.03	31.47	4.69	6.03

Although men donors were the most prevalent (*p* < 0.0001) in all districts, gender distribution throughout the districts did not statistically differ (X^2^ = 17.57, *p* = 0.4166) and was comparable to the pattern seen in governorates (Table 5).

**Table 5:** District distribution of blood donors (B.D.) according to gender in Lebanon. Men donors were the most prevalent (*p* < 0.0001) in all districts. Gender distribution throughout the districts did not statistically differ (X^2^ = 17.57, *p* = 0.4166).

	B.D. (%)	
	Female	Male
Akkar	13.96	86.04
Aley	19.34	80.66
Baabda	27.98	72.02
Baalbeck	20.65	79.35
Batroun	25.93	74.07
Beirut	23.11	76.89
Beqaa	19.96	80.04
Chouf	20.80	79.20
Jbeil	24.29	75.71
Kesserwan	27.15	72.85
Koura	20.32	79.68
Metn	26.76	73.24
Nabatieh	20.40	79.60
Sidon	16.10	83.90
Tripoli	15.62	84.38
Tyre	21.36	78.64
Zahle	19.68	80.32
Zgharta	26.29	73.71

## Discussion

Lebanon is distinguished by the absence of a governmental body that works on a Donor Management System to ensure continuous recruitment and scheduling of regular donors. This fragmented and decentralized blood system faces challenges in limited donors and blood shortage. Consequently, how ABO and RhD blood groups are distributed in the general population is of interest to improve blood services. The current research was performed over six years on 36,002 people throughout Lebanon to unravel the blood group distribution and perform rigorous blood donor demographics assessment. Our findings might pave the way for implementing improved decision-making strategies in Lebanon’s fragmented and decentralized blood management system.

Stakeholders such as ministries of health, non-governmental organizations that concentrate on global health, national transfusion services, and blood banks would better anticipate the necessary supply and prepare for sufficient transfusion services with a better knowledge of a country’s blood group distribution and blood requirements. Blood groups prevalence according to the ABO and RhD systems was initially studied in the Lebanese population. RhD+ groups were more widespread, with A+ being the most common, followed by the rest. RhD- groups were less common in the sequence A-, O-, B-, and AB-. There was no overall statistical significance in the frequency of blood types based on the ABO and RhD systems across governorates and districts. There were, however, significant geographical variations, with larger proportions of group B+ in the South, Nabatieh, and Baalbeck-Hermel. Except for the South and Baalbeck-Hermel, where the O+ group was more common, the A+ group predominated in the majority of governorates. In Batroun and Koura, the blood type AB-, which is the least common, was most frequent. Our findings on the regional diversity of blood group distribution would be helpful to design better recruitment strategies to prevent blood shortages.

Looking at the neighboring middle eastern countries, a large study from Jordan from 2013 to 2018 found that O and A were the most prevalent groups, followed by B and AB. The distribution of blood types O and A (37.44% and 36.82%, respectively) showed no significant differences [15]. In that research, the frequency rates of blood types B and AB were 18.62% and 7.12%, respectively, with B being comparable and AB being higher than previously reported in Saudi Arabia (3.9%) [16]. The prevalence of blood groups in Saudi Arabia was: A, 29.44%; B, 10.44%; AB, 1.15%; and O, 58.97% [16]. Furthermore, the prevalence of blood types in Syria’s general population was as follows: 40%, 8%, 47%, and 5%, respectively, in groups A, B, O, and AB [17]. In the United Arab Emirates [18] and Oman [19], a similar blood group distribution was found. In Iraq [20], this A and O preponderance were also seen. Interestingly, the A blood group in our study was predominant over the O group, which meets the blood groups prevalence seen in Turkey [21], in contrast to the rest of the Middle East countries where the O blood group is the most prevalent. Altogether, these percentages contrast from data in Asian countries such as India, where O group donors are most prevalent at 37.12%, followed by B at 32.26%, A at 22.88%, and AB being lowest at 7.74% [22]. Apart from India, the high prevalence of the B group is often observed in Asian countries such as Pakistan [23] and some ethnicities in China [7].

In-depth knowledge of a country’s blood requirements enables a more accurate forecast of blood supply and implementation of comprehensive transfusion services. In the present research, we studied the effect of gender and age on blood donation and performed a detailed mapping of blood donors’ groups according to the ABO and RhD systems over the entire country. Overall, similar blood groups distribution was noted in donors as the general prevalence of blood groups in Lebanon. Although the literature shows that donation rates tend to decrease above 65 years, the relative peak ages of donation differ globally. The age profile of blood donors shows that, proportionally, more young people donate blood in low- and middle-income countries than in high-income countries, which is in line with our current findings. In Europe, the age where donations are most common is in the 50-65 year range [24], whereas studies in other countries found the highest donor frequency in the age range of 18-25 years [25].

Furthermore, data about the gender profile of blood donors show that globally 33% of blood donations are given by women, although this ranges widely. Women were less likely to donate blood, especially in Southern and Eastern Europe, but this gender gap has declined [24]. In China, compared to the United States, donations are made by younger donors, and donors give infrequently; however, women composed around half of the donors [26]. Women often do not significantly contribute to blood donation in middle eastern countries and patriarchal societies due to societal factors, misconceptions, and religion [15]. Our results showed that the percentage of female donors was 23.72%, much higher than the previously reported 2% in Lebanon [27]. This discrepancy might reside in the targeted population of blood donors in our study since the DSC non-governmental organization usually conducts awareness sessions and blood donation campaigns (blood drives) in schools, universities, and public spaces, whereas the prior study was conducted using a database from hospital blood banks. These new results emphasize the critical role of non-governmental organizations in increasing the awareness of voluntary non-remunerated blood donations in a fragmented decentralized blood system such as in Lebanon and highlight the role of women whilst busting the myths/taboos gravitating around their donations. Strikingly, there was no difference in female donor percentage between the different governorates and districts. Since the country’s establishment, Lebanon has had sectarianism deeply rooted in its political and social systems. Even though a particular mosaic exists across the country when religion is considered, clear delimitations still reside between the geographical regions, with Mount Lebanon being mostly Christian and Druze, the North and Beirut being mostly Sunni, and the South and North Beqaa being mostly Shiite [28]. However, the comparable percentages of female donors between the regions mitigate the disparities in the societies and further ascertain the significance of the work conducted by non-governmental organizations such as DSC.

The majority of participants in the current study contributed at least once, with 55.09% contributing just once and 91.79% donating no more than five times. Blood donation loyalty evolves throughout a person’s life as a consequence of many experiences. Events in one’s life may impact one’s participation in a prosocial activity, perhaps as a consequence of the loss of one’s human and social capital [9]. Add to that, a significant deterrent to blood donation was a person’s negative impression of his or her health, whereas a healthy lifestyle is associated positively with blood donation [29]. Consequently, regular blood donors are essential to maintaining the safety of blood supplies. First-time blood donors may be encouraged to give again by adopting targeted interventions such as emotional letters, instructional letters, and phone reminders that enhance the return rate of first-time donors, which can increase the number of donors [30].

Keeping a sustainable and healthy blood supply that can satisfy clinical demand is one of the most significant difficulties in underdeveloped nations. Overall, the Lebanese blood transfusion system is decentralized and fragmented due to the tremendous but unevenly distributed private sector, especially hospitals [2, 10-13]. NGOs, including the Lebanese Red Cross and DSC association, support a healthy blood supply by providing essential resources. Nevertheless, Lebanon is still several roadblocks away from WHO’s target of 100% voluntary non-remunerated blood donation by 2025. Furthermore, many still have misconceptions about giving blood, such as the fear of getting infectious illnesses from contaminated needles and developing anemia. To exacerbate the situation, minor efforts have been exerted by public health officials to work towards creating an effective blood transfusion system, and the few issued initiatives were not adequately implemented. Because of this, it is crucial to understand how ABO and RhD blood types are distributed in the general Lebanese population and to exhaustively dissect blood donors’ behavior to ensure efficient blood services.

Several limitations of our study might be identified. To begin with, all attempts to collect blood through blood drives were managed by the DSC non-governmental organization, which was not readily supported by hospital blood banks. In Lebanon’s fragmented blood system, blood banks operate autonomously with no clear or unified national direction, which may have led to hospitals’ limited involvement. Furthermore, data input was not a priority for DSC when they first began, therefore a large quantity of vital data was lost in the analysis and triage. To make matters worse, individuals from rural areas were not as numerous as those from urban areas, owing to the tremendous increase in urbanization at the cost of rural areas. Finally, religious and societal attitudes had a role in certain areas’ low participation rates.

## Conclusions

With this six-year retrospective research on blood type prevalence and demographics of blood donors in Lebanon, it is possible to chart new paths toward a more coherent and unified blood management system in the nation. The report also emphasizes the significant efforts made by non-governmental organizations to act as surrogates for the country’s inadequate official initiatives in order to provide a continuous supply of blood for the population.

These findings also provide important clinical information that will be useful in the development of future transfusion medicine policies and approaches in the country, which may be especially important in a region that is prone to socio-political conflicts culminating in humanitarian crises, with the escalation of blood demand often coinciding with such crises.
